# Supplementary material for: Can random walking on a Hi-C contact matrix lead to data quality improvement? An assessment
Source: PLoS One. 2025 Sep 23;20(9):e0327100. doi: 10.1371/journal.pone.0327100 (PMC12456815; doi:10.1371/journal.pone.0327100)
Supplement: S1 Table — (DOCX) [file pone.0327100.s001.docx]

**S1 Table. Summary of Hi-C data improvement methods**$\boldsymbol{.}^{a}$

| Method | Citation | Data Type | Imputation Approach |
| --- | --- | --- | --- |
| GenomeDISCO | Ursu et al., 2018 [16] | Bulk | RWS |
| scHiCluster | Zhou et al., 2019 [12] | Single-cell | Linear convolution + RWR |
| ScHiC-Rep | Zhen et al., 2021 [19] | Single-cell | Linear convolution + RWR + Graph convolution network |
| SnapHiC | Yu et al., 2021 [20] | Single-cell | RWR |
| SnapHiC2 | Li et al, 2022 [22] | Single-cell | Sliding window approach to approximate RWR |
| scHiCStackL | Wu et al., 2022 [22] | Single-cell | Linear convolution + RWR |
| scHiCPTR | Lyu et al., 2022 [25] | Single-cell | Linear convolution + RWR |
| HiCRep | Yang et al., 2017 [14] | Bulk | 2D mean filter (aka kernel smoothing, linear convolution) |
| HiCPlus | Zhang et al., 2018 [15] | Bulk | Deep convolutional neural network |
| SCL | Zhu & Wang, 2019 [17] | Single-cell | 2D Gaussian kernel (aka kernel smoothing) |
| DeepHiC | Hong et al., 2020 [18] | Bulk | Convolutional residual network |
| Higashi | Zhang et al., 2022 [23] | Single-cell | Hypergraph neural network |
| HiCImpute | Xie et al., 2022 [24] | Single-cell | Bayesian hierarchical network |

${}^{a}$The top block consists of methods that are random-walk-related, while the bottom block consists of other methods.
