# Supplementary material for: Can random walking on a Hi-C contact matrix lead to data quality improvement? An assessment
Source: PLoS One. 2025 Sep 23;20(9):e0327100. doi: 10.1371/journal.pone.0327100 (PMC12456815; doi:10.1371/journal.pone.0327100)
Supplement: S2 Table — (DOCX) [file pone.0327100.s002.docx]

**S2 Table. Summary of three TAD detection algorithms.**

| Method | Citation | Version | Parameter setting${}^{a}$ |
| --- | --- | --- | --- |
| CaTCH | Zhan et al., 2017 [28] | V1.0 | Reciprocal insulation (RI) threshold: 0.65 |
| HiCseg | Levy-Leduc et al., 2014 [29] | V1.12.2 | Count matrix: extended block-diagonal model and Poisson distribution; Normalized/smoothed matrix: extended block-diagonal model and Gaussian distribution |
| TopDom | Shin et al., 2016 [30] | V0.10.1 | Window size: 5 |

${}^{a}$These values were recommended in the original papers and used in our analyses throughout.
