# Supplementary material for: Can random walking on a Hi-C contact matrix lead to data quality improvement? An assessment
Source: PLoS One. 2025 Sep 23;20(9):e0327100. doi: 10.1371/journal.pone.0327100 (PMC12456815; doi:10.1371/journal.pone.0327100)
Supplement: S1 Fig — Heatmaps demonstrating the behavior of random walk methods. Heatmap visualization of a normalized contact matrix P given in the form of Equation (1) (first plot in the 1st row), RWS-smoothed matrices with s= 2, 3, and 10 steps (second to the fourth plots in the 1st row), and RWR-smoothed matrices with restart probability α= 0.05, 0.1, 0.2, and 0.5 (2nd row). The total number of bins is chosen to be N=200, and the number of TADs k=5. The domain sizes ni are specified as 50, 30, 20, 90, and 10, respectively. The magnitude of outside-domain interaction frequency d=12N=0.0025. The color scheme ranges from 0 (white) to 0.0525 (red, the maximum value in P), with those smoothed counts greater than 0.0525 capped at 0.0525 (e.g., all the main diagonals in the RWR-smoothed matrices). (DOCX) [file pone.0327100.s003.docx]

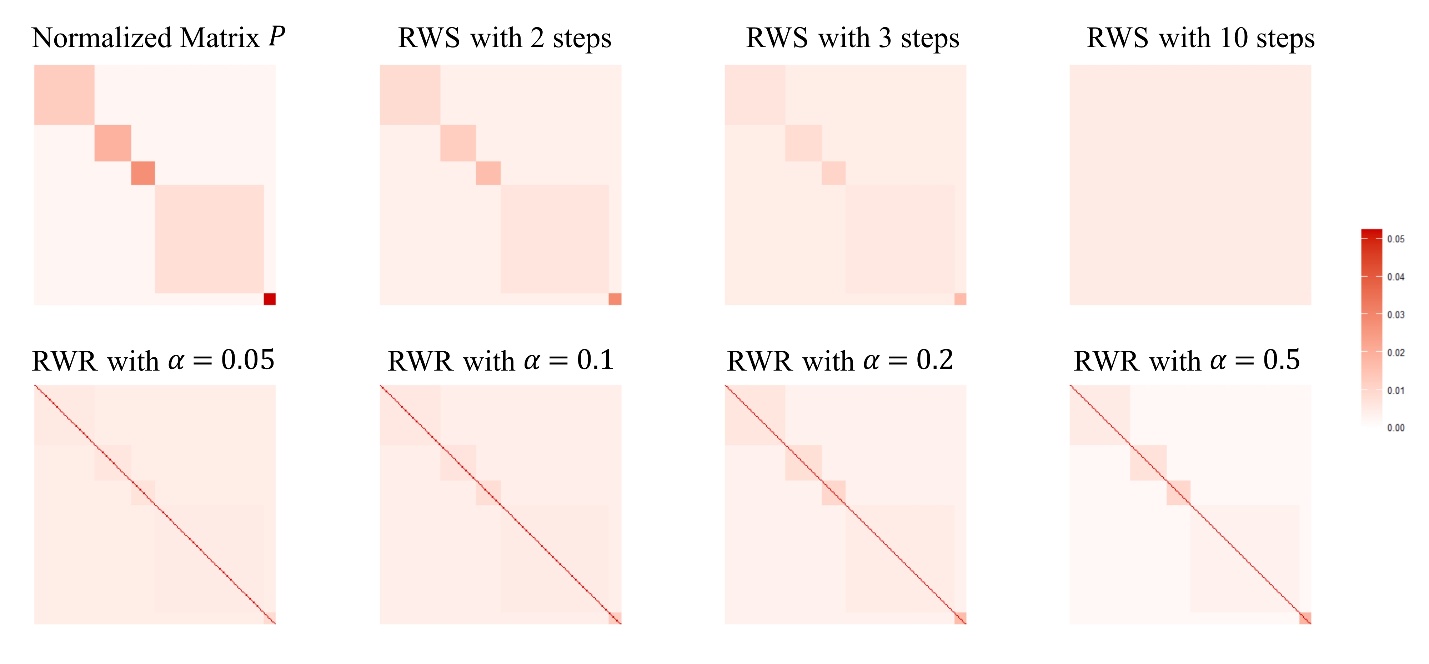


**S1 Fig**. **Heatmaps demonstrating the behavior of random walk methods.** Heatmap visualization of a normalized contact matrix $P$ given in the form of Equation $(1)$ (first plot in the 1^st^ row), RWS-smoothed matrices with $s=$ 2, 3, and 10 steps (second to the fourth plots in the 1^st^ row), and RWR-smoothed matrices with restart probability $\alpha=$ 0.05, 0.1, 0.2, and 0.5 (2^nd^ row). The total number of bins is chosen to be $N=200$, and the number of TADs $k=5$. The domain sizes $n_{i}$ are specified as 50, 30, 20, 90, and 10, respectively. The magnitude of outside-domain interaction frequency $d=\frac{1}{2N}=0.0025$. The color scheme ranges from 0 (white) to 0.0525 (red, the maximum value in $P$), with those smoothed counts greater than 0.0525 capped at 0.0525 (e.g., all the main diagonals in the RWR-smoothed matrices).
