# Supplementary material for: Can random walking on a Hi-C contact matrix lead to data quality improvement? An assessment
Source: PLoS One. 2025 Sep 23;20(9):e0327100. doi: 10.1371/journal.pone.0327100 (PMC12456815; doi:10.1371/journal.pone.0327100)
Supplement: S2 Fig — RWS-smoothed data and TAD detection results for an idealized dataset. Heatmap visualization of the RWS-smoothed matrices (with s= 2, 3, 4, 5, and 10) in Simulation Study 1, along with the detected domain boundaries and ARI values (bottom left corner). The total number of bins is N=200. The number of TADs k=5, with sizes ni= 50, 30, 20, 90 and 10, respectively. Same layout as in Fig 1. The color scheme for all the heatmaps ranges from 0 (white) to 0.05 (red). (DOCX) [file pone.0327100.s004.docx]

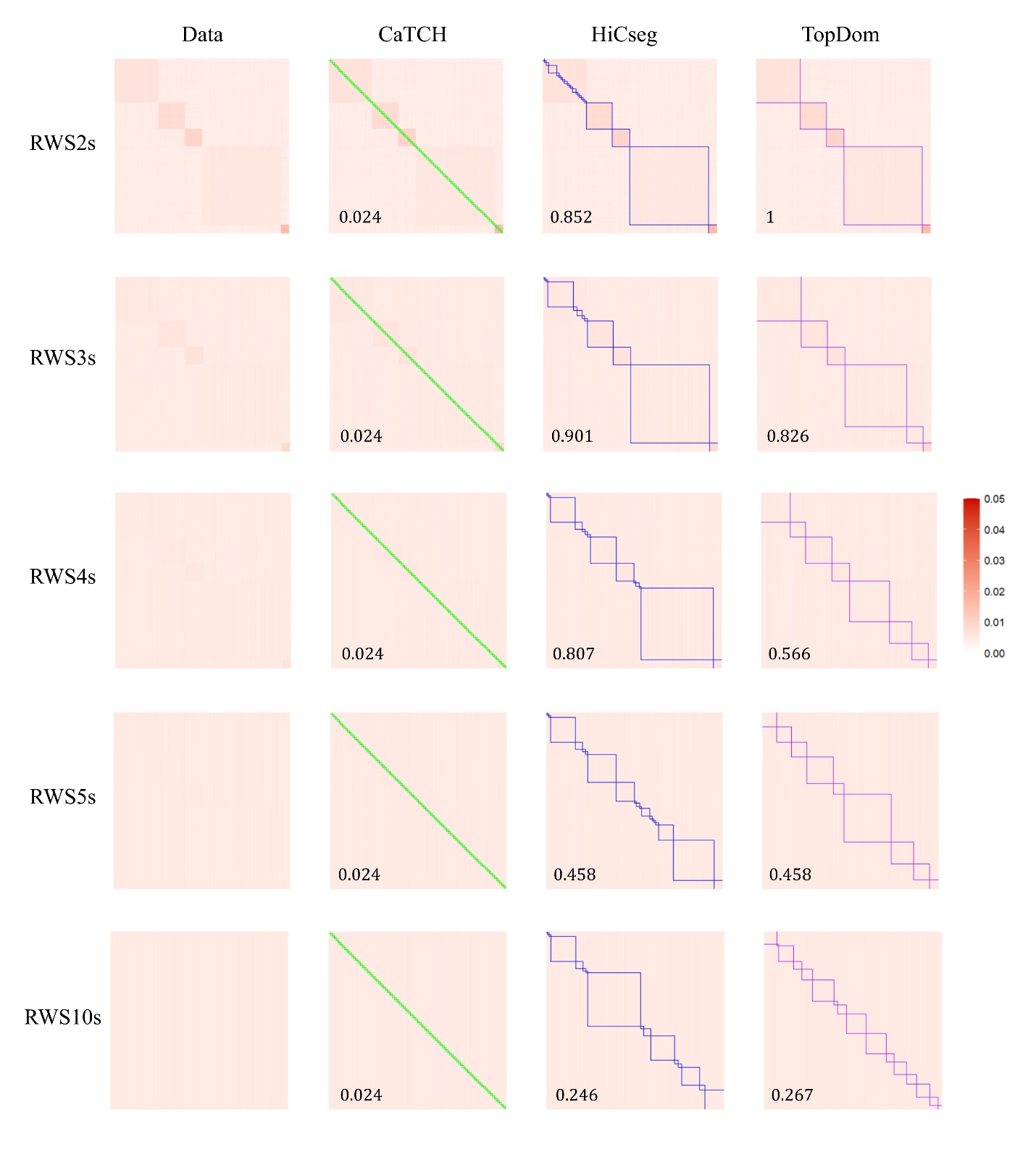


**S2 Fig.** **RWS-smoothed data and TAD detection results for an idealized dataset.** Heatmap visualization of the RWS-smoothed matrices (with $s=$ 2, 3, 4, 5, and 10) in Simulation Study 1, along with the detected domain boundaries and ARI values (bottom left corner). The total number of bins is $N=200$. The number of TADs $k=5$, with sizes $n_{i}=$ 50, 30, 20, 90 and 10, respectively. Same layout as in Fig 1. The color scheme for all the heatmaps ranges from 0 (white) to 0.05 (red).
