# Supplementary material for: Can random walking on a Hi-C contact matrix lead to data quality improvement? An assessment
Source: PLoS One. 2025 Sep 23;20(9):e0327100. doi: 10.1371/journal.pone.0327100 (PMC12456815; doi:10.1371/journal.pone.0327100)
Supplement: S3 Fig — RWR-smoothed data and TAD detection results for an idealized dataset. Heatmap visualization of the RWR-smoothed matrices (with α= 0.05, 0.1, 0.2, and 0.5) in Simulation Study 1, along with the detected domain boundaries and ARI values (bottom left corner). The total number of bins is N=200. The number of TADs k=5, with sizes ni= 50, 30, 20, 90 and 10, respectively. Same layout as in Fig 1. The color scheme for all the heatmaps ranges from 0 (white) to 0.05 (red), with those values that are greater than 0.05 capped at 0.05. (DOCX) [file pone.0327100.s005.docx]

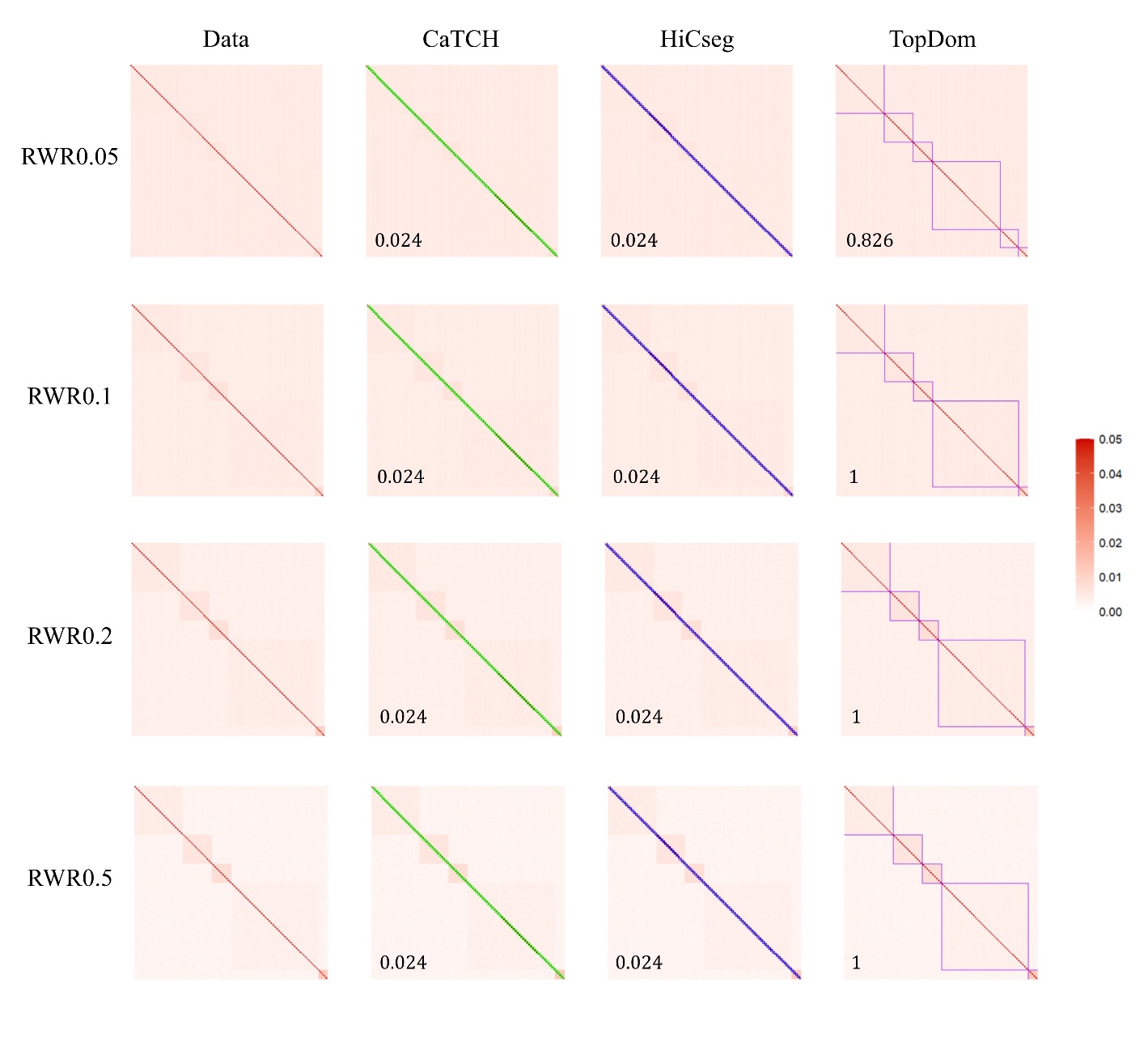


**S3 Fig.** **RWR-smoothed data and TAD detection results for an idealized dataset.** Heatmap visualization of the RWR-smoothed matrices (with $\alpha=$ 0.05, 0.1, 0.2, and 0.5) in Simulation Study 1, along with the detected domain boundaries and ARI values (bottom left corner). The total number of bins is $N=200$. The number of TADs $k=5$, with sizes $n_{i}=$ 50, 30, 20, 90 and 10, respectively. Same layout as in Fig 1. The color scheme for all the heatmaps ranges from 0 (white) to 0.05 (red), with those values that are greater than 0.05 capped at 0.05.
