# Supplementary material for: Can random walking on a Hi-C contact matrix lead to data quality improvement? An assessment
Source: PLoS One. 2025 Sep 23;20(9):e0327100. doi: 10.1371/journal.pone.0327100 (PMC12456815; doi:10.1371/journal.pone.0327100)
Supplement: S4 Fig — Observed count matrix along with RWS-smoothed data and TAD detection results for a simulated dataset based on biophysical law. Heatmap visualization of the simulated count matrix E (first row) and the RWS-smoothed matrices (with s= 2, 3, 4, 5, and 10, for the second to the sixth rows, respectively) in one realization of the simulation procedure described in Simulation Study 2, with the same layout as in Fig 1. The color scheme for the simulated count matrix E heatmap ranges from 0 (white) to 30 (red), where 30 is the 99.5-th percentile of the simulated count. The color scheme for the heatmaps of the RWS-smoothed matrices ranges from 0 (white) to 0.007 (red). (DOCX) [file pone.0327100.s006.docx]

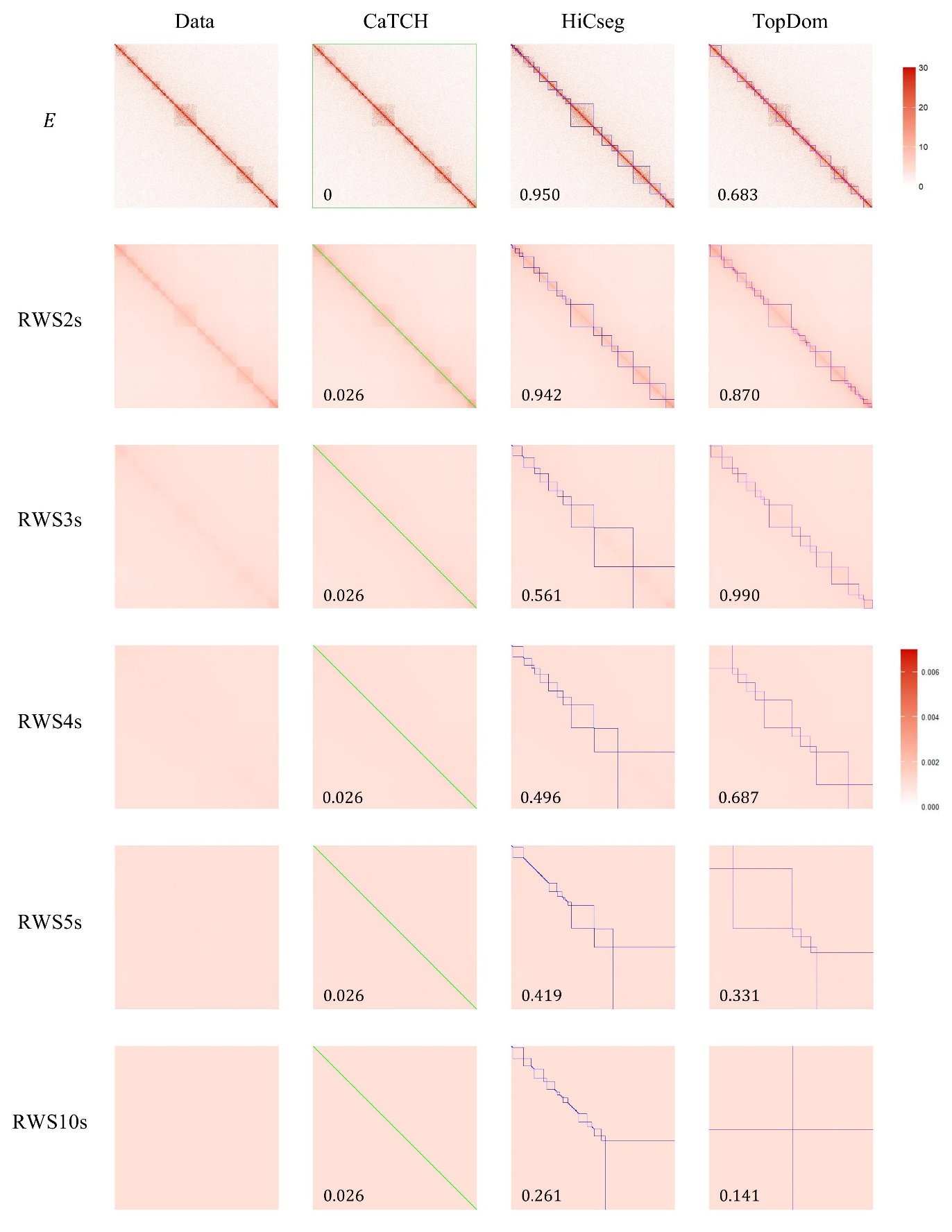


**S4 Fig.** **Observed count matrix along with** **RWS-smoothed data and TAD detection results for a simulated dataset based on biophysical law.** Heatmap visualization of the simulated count matrix $E$ (first row) and the RWS-smoothed matrices (with $s=$ 2, 3, 4, 5, and 10, for the second to the sixth rows, respectively) in one realization of the simulation procedure described in Simulation Study 2, with the same layout as in Fig 1. The color scheme for the simulated count matrix $E$ heatmap ranges from 0 (white) to 30 (red), where 30 is the 99.5-th percentile of the simulated count. The color scheme for the heatmaps of the RWS-smoothed matrices ranges from 0 (white) to 0.007 (red).
