# Supplementary material for: Can random walking on a Hi-C contact matrix lead to data quality improvement? An assessment
Source: PLoS One. 2025 Sep 23;20(9):e0327100. doi: 10.1371/journal.pone.0327100 (PMC12456815; doi:10.1371/journal.pone.0327100)
Supplement: S5 Fig — KR-normalized observed count matrix along with RWR-smoothed data and TAD detection results for a simulated dataset based on biophysical law. Heatmap visualization of the KR-normalized matrix EKR (first row) and the RWR-smoothed matrices (with α= 0.05, 0.1, 0.2, and 0.5, for the second to the fifth rows, respectively) in one realization of the simulation procedure described in Simulation Study 2, with the same layout as in Fig 1. The color scheme for all the heatmaps ranges from 0 (white) to 0.007 (red), with those values that are greater than 0.007 capped at 0.007. (DOCX) [file pone.0327100.s007.docx]

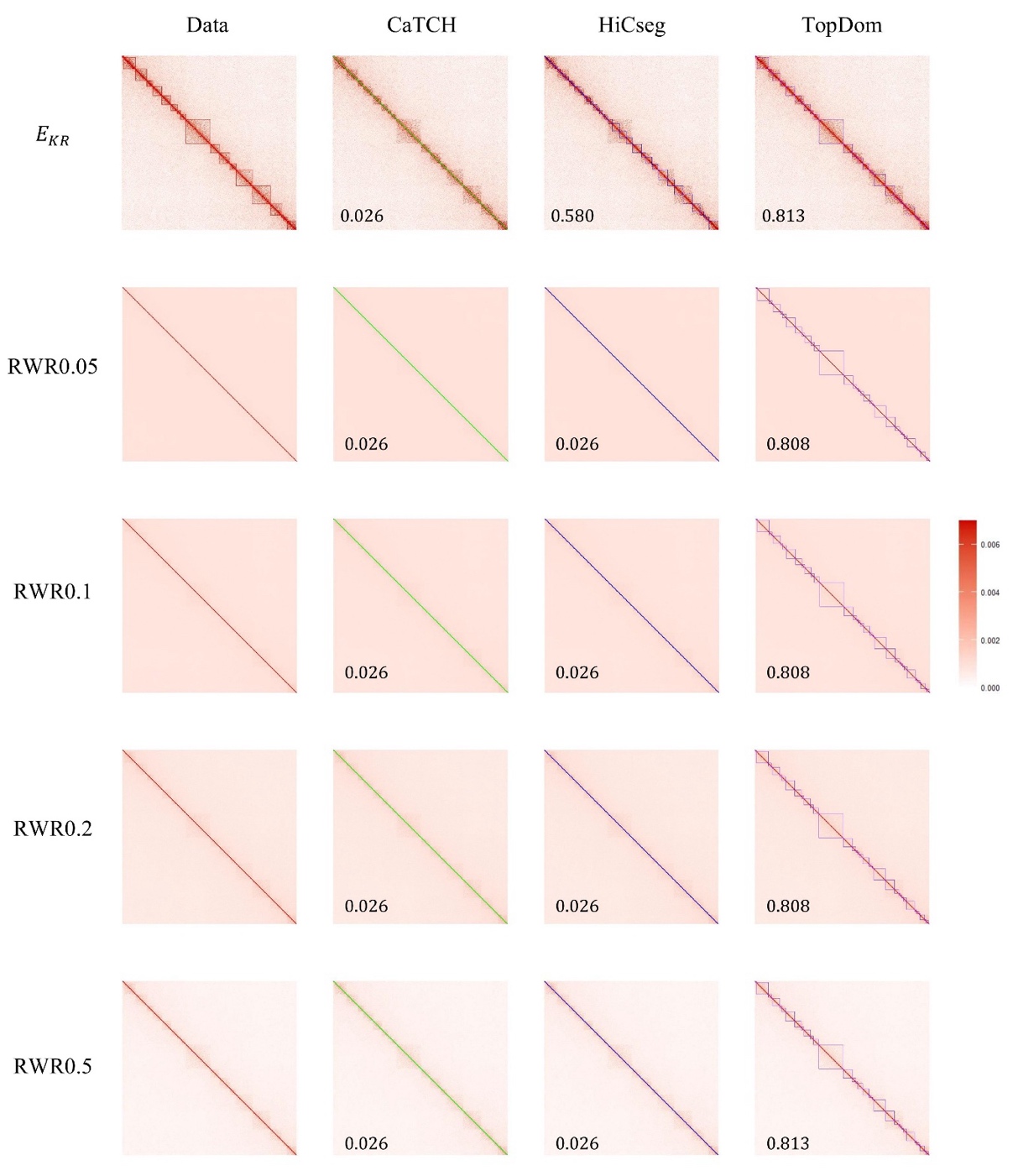


**S5 Fig**. **KR-normalized** **observed count matrix along with** **RWR-smoothed data and TAD detection results for a simulated dataset based on biophysical law.** Heatmap visualization of the KR-normalized matrix $E_{KR}$ (first row) and the RWR-smoothed matrices (with $\alpha=$ 0.05, 0.1, 0.2, and 0.5, for the second to the fifth rows, respectively) in one realization of the simulation procedure described in Simulation Study 2, with the same layout as in Fig 1. The color scheme for all the heatmaps ranges from 0 (white) to 0.007 (red), with those values that are greater than 0.007 capped at 0.007.
