# Supplementary material for: Can random walking on a Hi-C contact matrix lead to data quality improvement? An assessment
Source: PLoS One. 2025 Sep 23;20(9):e0327100. doi: 10.1371/journal.pone.0327100 (PMC12456815; doi:10.1371/journal.pone.0327100)
Supplement: S6 Fig — Data and TAD detection results for the subsampled data based on a K562 bulk dataset. (a) Heatmap of the bulk count matrix (first row), the KR normalized bulk matrix (third row), one realization of the subsampled matrix (second row) and the KR normalized subsampled matrix (fourth row) with the detected TAD boundaries. The ARI value of each detected boundary on the subsampled/KR-normalized matrix (compared to the one detected on the bulk matrix) is listed at the bottom left corner of the heatmap. The color scheme for the bulk matrix heatmap ranges from 0 (white) to 10 (red), with those values that are greater than 10 capped at 10. The color scheme for the subsampled matrix heatmap ranges from 0 (white) to 2 (red), with those values that are greater than 2 capped at 2. The color scheme for the heatmaps of the KR-normalized matrices ranges from 0 (white) to 0.05 (red), with those values that are greater than 0.05 capped at 0.05. (b) Violin plots for the ARI values of CaTCH (first row) and HiCseg (second row) domain boundaries with 100 realizations of the subsampling procedure described in Simulation Study 3. (DOCX) [file pone.0327100.s008.docx]

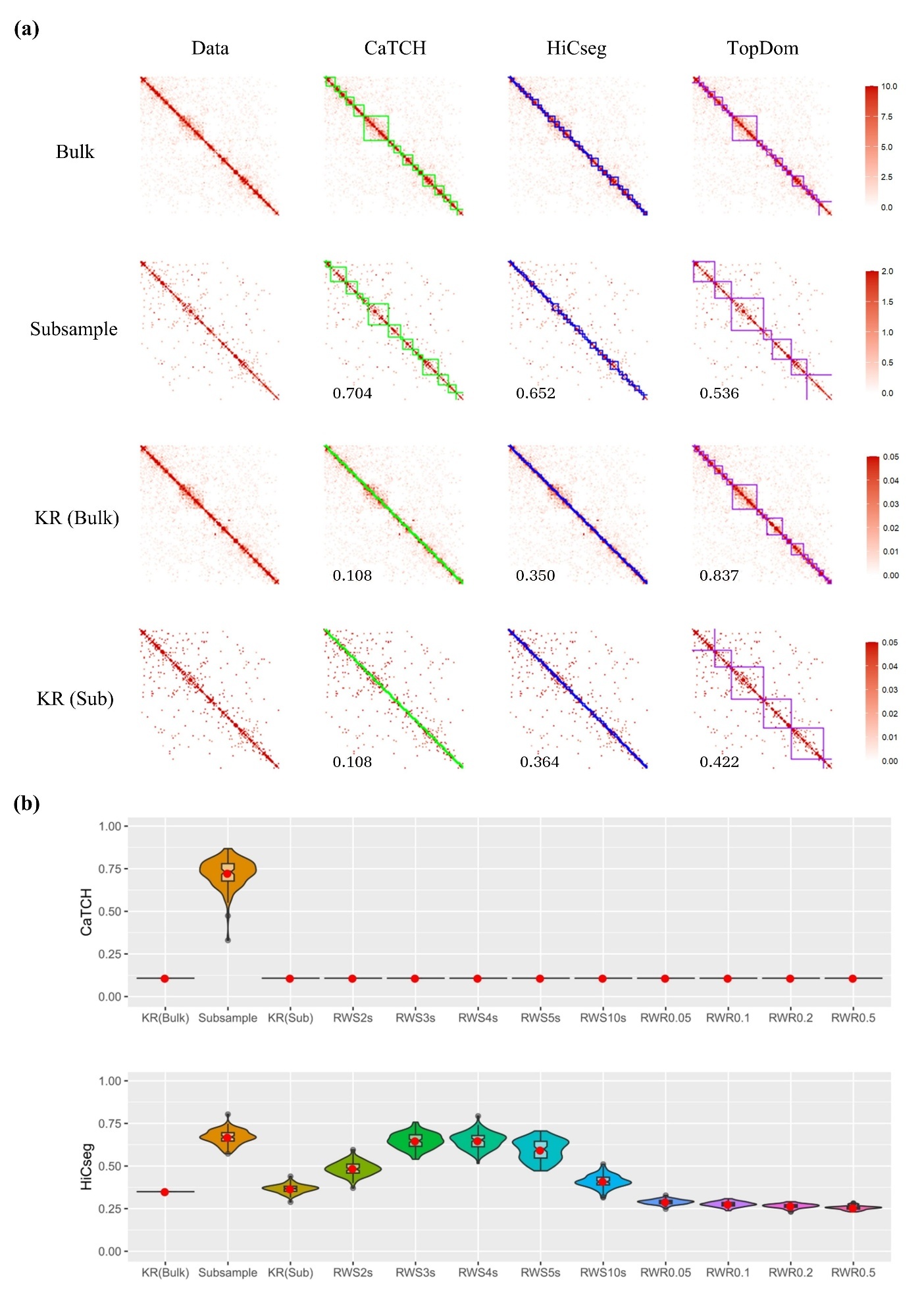


**S6 Fig.** **Data and TAD detection results for the subsampled data based on a K562 bulk dataset.** (a) Heatmap of the bulk count matrix (first row), the KR normalized bulk matrix (third row), one realization of the subsampled matrix (second row) and the KR normalized subsampled matrix (fourth row) with the detected TAD boundaries. The ARI value of each detected boundary on the subsampled/KR-normalized matrix (compared to the one detected on the bulk matrix) is listed at the bottom left corner of the heatmap. The color scheme for the bulk matrix heatmap ranges from 0 (white) to 10 (red), with those values that are greater than 10 capped at 10. The color scheme for the subsampled matrix heatmap ranges from 0 (white) to 2 (red), with those values that are greater than 2 capped at 2. The color scheme for the heatmaps of the KR-normalized matrices ranges from 0 (white) to 0.05 (red), with those values that are greater than 0.05 capped at 0.05. (b) Violin plots for the ARI values of CaTCH (first row) and HiCseg (second row) domain boundaries with 100 realizations of the subsampling procedure described in Simulation Study 3.
