# Supplementary material for: Can random walking on a Hi-C contact matrix lead to data quality improvement? An assessment
Source: PLoS One. 2025 Sep 23;20(9):e0327100. doi: 10.1371/journal.pone.0327100 (PMC12456815; doi:10.1371/journal.pone.0327100)
Supplement: S7 Fig — RWS-smoothed data and TAD detection results for a subsample based on a K562 bulk dataset. Heatmaps and identified boundaries on the RWS-smoothed matrices in one realization of the subsampling procedure described in Section 3.1.3, with the same layout as in Fig 3(a). The color scheme for all the heatmaps ranges from 0 (white) to 0.05 (red), with those values that are greater than 0.05 capped at 0.05. (DOCX) [file pone.0327100.s009.docx]

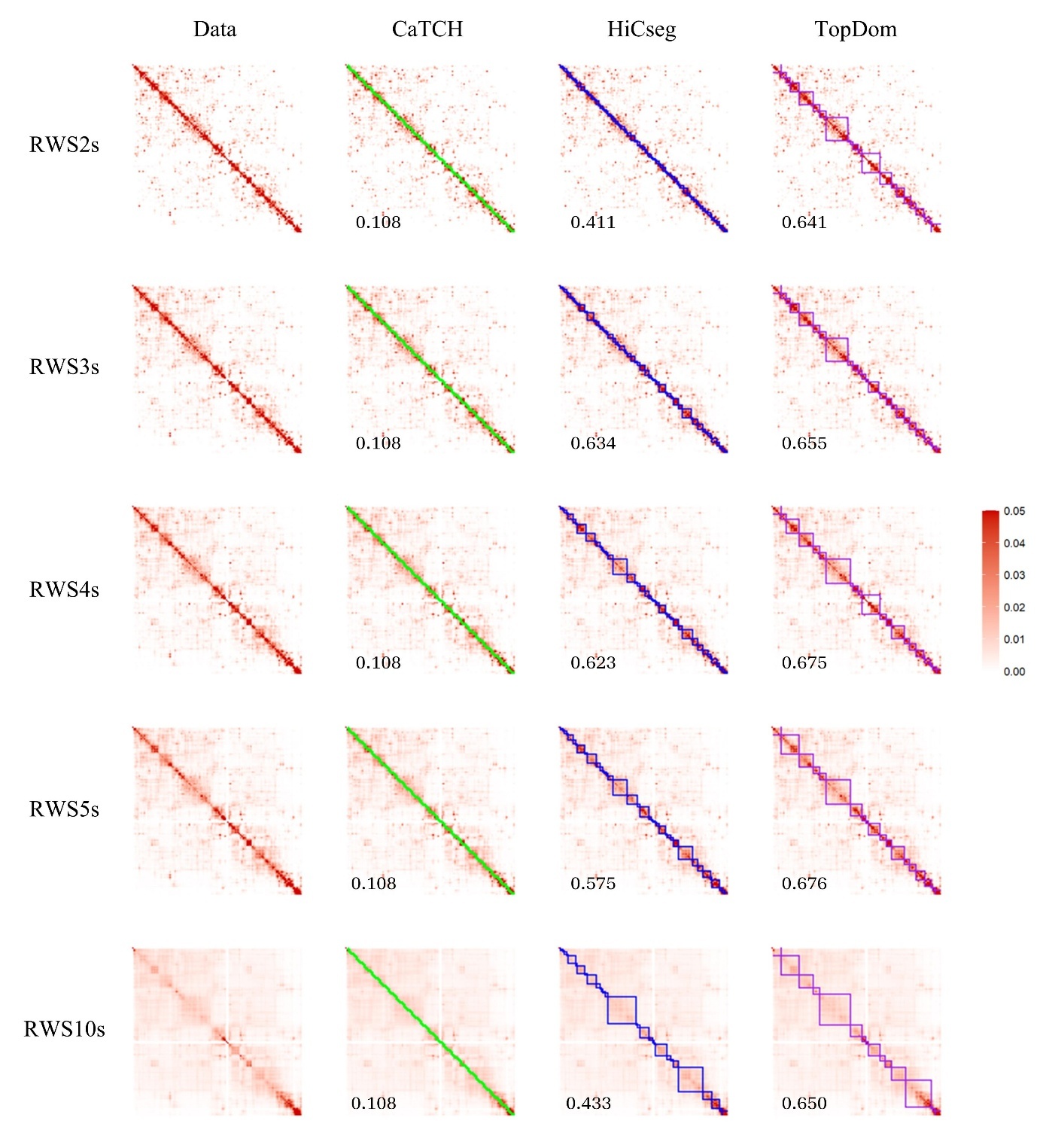


**S7 Fig**. **RWS-smoothed data and TAD detection results for a subsample based on a K562 bulk dataset.** Heatmaps and identified boundaries on the RWS-smoothed matrices in one realization of the subsampling procedure described in Section 3.1.3, with the same layout as in Fig 3(a). The color scheme for all the heatmaps ranges from 0 (white) to 0.05 (red), with those values that are greater than 0.05 capped at 0.05.
