# Supplementary material for: Can random walking on a Hi-C contact matrix lead to data quality improvement? An assessment
Source: PLoS One. 2025 Sep 23;20(9):e0327100. doi: 10.1371/journal.pone.0327100 (PMC12456815; doi:10.1371/journal.pone.0327100)
Supplement: S9 Fig — The heatmaps and identified boundaries on the bulk count matrix and RWS-smoothed matrices of hESC data. The ARI value of each detected boundary on the RWS-smoothed matrix (compared to the one detected on the count matrix) is listed at the bottom left corner of the heatmap. The color scheme for the bulk matrix heatmap ranges from 0 (white) to 150 (red), with those values that are greater than 150 capped at 150. The color scheme for all the other heatmaps ranges from 0 (white) to 0.05 (red), with those values that are greater than 0.05 capped at 0.05. (DOCX) [file pone.0327100.s011.docx]

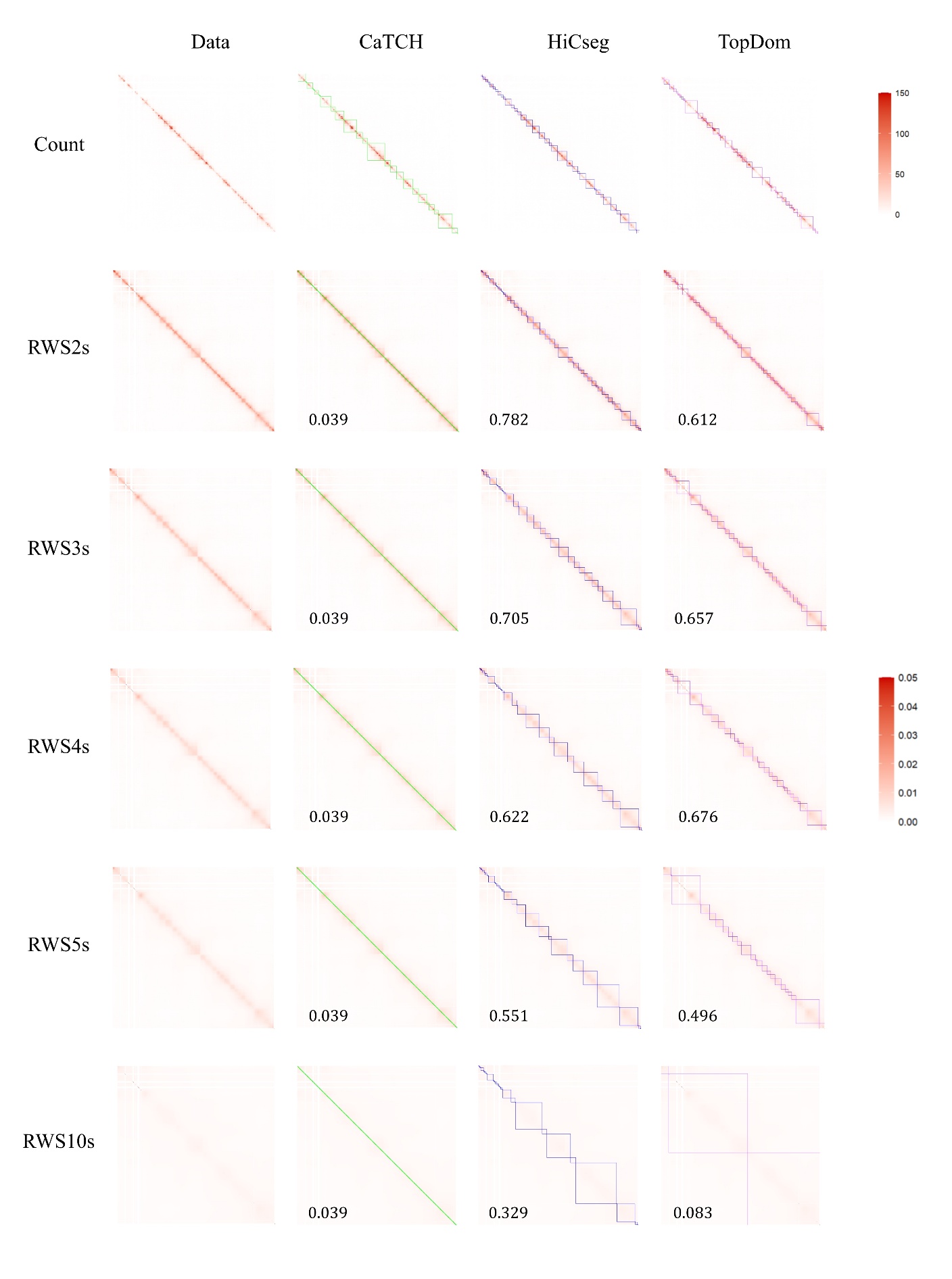


**S9 Fig**. **The heatmaps and identified boundaries on the bulk count matrix and RWS-smoothed matrices of hESC data.** The ARI value of each detected boundary on the RWS-smoothed matrix (compared to the one detected on the count matrix) is listed at the bottom left corner of the heatmap. The color scheme for the bulk matrix heatmap ranges from 0 (white) to 150 (red), with those values that are greater than 150 capped at 150. The color scheme for all the other heatmaps ranges from 0 (white) to 0.05 (red), with those values that are greater than 0.05 capped at 0.05.
