# Supplementary material for: Can random walking on a Hi-C contact matrix lead to data quality improvement? An assessment
Source: PLoS One. 2025 Sep 23;20(9):e0327100. doi: 10.1371/journal.pone.0327100 (PMC12456815; doi:10.1371/journal.pone.0327100)
Supplement: S10 Fig — Data and results on a bulk hESC dataset. This figure provides the heatmaps and identified boundaries on the KR normalized and RWR-smoothed bulk matrices of hESC data described The ARI value of each detected boundary on the KR-normalized/RWR-smoothed matrix (compared to the one detected on the count matrix) is listed at the bottom left corner of the heatmap. The color scheme for all the heatmaps ranges from 0 (white) to 0.05 (red), with those values that are greater than 0.05 capped at 0.05. (DOCX) [file pone.0327100.s012.docx]

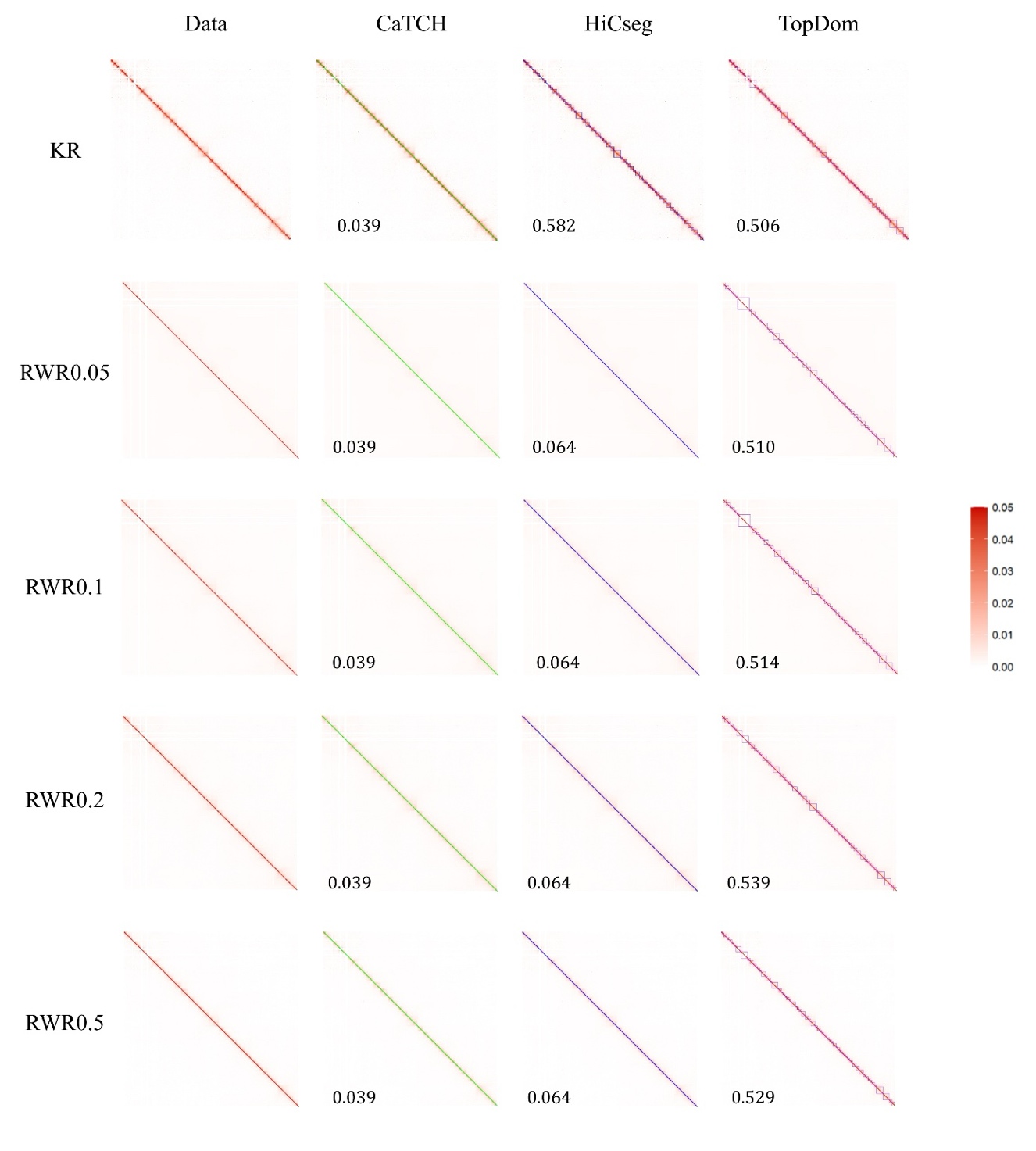


**S10 Fig**. **Data and results on a bulk hESC dataset.** This figure provides the heatmaps and identified boundaries on the KR normalized and RWR-smoothed bulk matrices of hESC data described The ARI value of each detected boundary on the KR-normalized/RWR-smoothed matrix (compared to the one detected on the count matrix) is listed at the bottom left corner of the heatmap. The color scheme for all the heatmaps ranges from 0 (white) to 0.05 (red), with those values that are greater than 0.05 capped at 0.05.
