# Supplementary material for: Can random walking on a Hi-C contact matrix lead to data quality improvement? An assessment
Source: PLoS One. 2025 Sep 23;20(9):e0327100. doi: 10.1371/journal.pone.0327100 (PMC12456815; doi:10.1371/journal.pone.0327100)
Supplement: S11 Fig — Data and results on a single cell dataset. Heatmap visualization of the composite GM count matrix (1st row, by summing up all 14 GM cells), count matrix of one GM cell (2nd row, GSM3314359), the composite PBMC count matrix (3rd row, by summing up all 18 GM cells), count matrix of one PBMC cell (4th row, GSM3314376). The ARI value was calculated between the composite and single-cell count data within the same TAD finding algorithm and listed at the bottom left corner of the heatmap of a single-cell count matrix. (DOCX) [file pone.0327100.s013.docx]

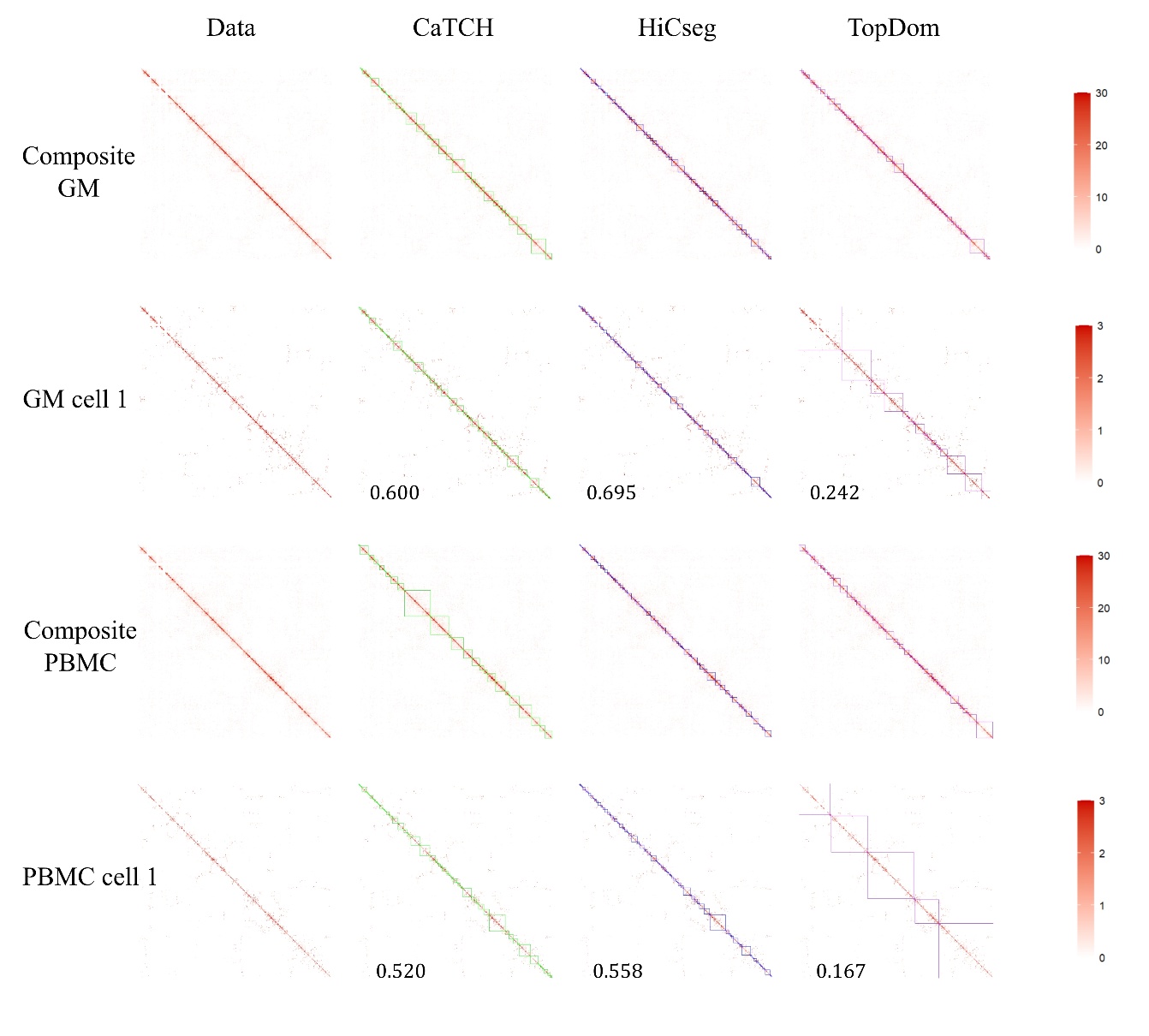


**S11 Fig**. **Data and results on a single cell dataset.** Heatmap visualization of the composite GM count matrix (1^st^ row, by summing up all 14 GM cells), count matrix of one GM cell (2^nd^ row, GSM3314359), the composite PBMC count matrix (3^rd^ row, by summing up all 18 GM cells), count matrix of one PBMC cell (4^th^ row, GSM3314376). The ARI value was calculated between the composite and single-cell count data within the same TAD finding algorithm and listed at the bottom left corner of the heatmap of a single-cell count matrix.
