# Supplementary material for: Can random walking on a Hi-C contact matrix lead to data quality improvement? An assessment
Source: PLoS One. 2025 Sep 23;20(9):e0327100. doi: 10.1371/journal.pone.0327100 (PMC12456815; doi:10.1371/journal.pone.0327100)
Supplement: S12 Fig — Influence of sparsity on random walk smoothing methods and TAD detection results. The subsampling procedure described in Simulation Study 3 (Section 3.1.3) was repeated 100 times for various subsampling percentages, ranging from 5% to 50% with an increment of 5%, and the median ARI is plotted: CaTCH (top row), HiCseg (middle row), and TopDom (bottom row). (DOCX) [file pone.0327100.s014.docx]

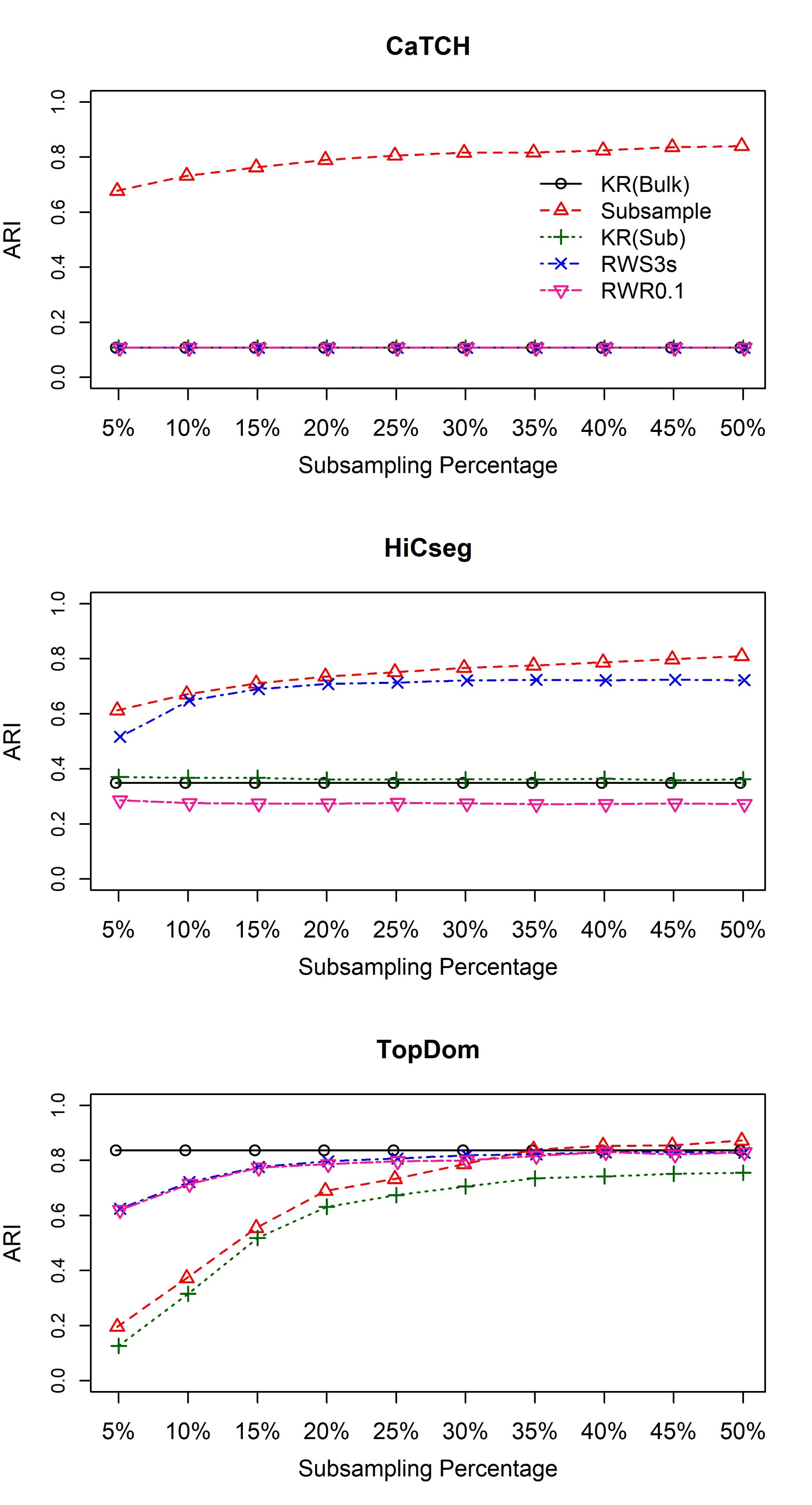


**S12 Fig**. **Influence of sparsity on random walk smoothing methods and TAD detection results.** The subsampling procedure described in Simulation Study 3 (Section 3.1.3) was repeated 100 times for various subsampling percentages, ranging from 5% to 50% with an increment of 5%, and the median ARI is plotted: CaTCH (top row), HiCseg (middle row), and TopDom (bottom row).
